# Epidemiological assessment of 5598 brucellosis inpatients in Spain (1997–2015)

**DOI:** 10.1017/S0950268821001151

**Published:** 2021-05-14

**Authors:** Beatriz Rodríguez-Alonso, Hugo Almeida, Montserrat Alonso-Sardón, Virginia Velasco-Tirado, Ángela Romero-Alegria, Javier Pardo-Lledias, Amparo López-Bernus, José Luis Pérez Arellano, Moncef Belhassen-García

**Affiliations:** 1 Servicio de Medicina Interna, Complejo Asistencial Universitario de Salamanca (CAUSA), Centro de Investigación de Enfermedades Tropicales de la Universidad de Salamanca (CIETUS), Instituto de Investigación Biomédica de Salamanca (IBSAL), Salamanca, España; 2 Medicina Interna, Unidade Local de Saude de Guarda, Guarda, Portugal; 3 Conselho Nacional do Médico Interno – Orden dos Médicos, Lisbon, Portugal; 4 Area of Preventive Medicine and Public Health, Instituto de investigación biomédica de Salamanca (IBSAL), CIETUS, University of Salamanca, Salamanca, Spain; 5 Servicio de Dermatología, CAUSA, CIETUS, IBSAL, Salamanca, España; 6 Servicio de Medicina Interna, CAUSA, IBSAL, CIETUS, Salamanca, Spain; 7 Servicio de Medicina Interna, Hospital Marques de Valdecilla, CIETUS, Avenida Valdecilla S/N, Santander, Spain; 8 Servicio de Medicina Interna, Infectious Diseases Section, CAUSA, IBSAL, CIETUS, University of Salamanca, Salamanca, Spain; 9 Infectious Diseases and Tropical Medicine Unit, Complejo Hospitalario Universitario Insular-Materno Infantil de Gran Canaria, Las Palmas de Gran Canaria, Spain; 10 Servicio de Medicina Interna. Unidad de Enfermedades Infecciosas, CAUSA, IBSAL, CIETUS, Universidad de Salamanca, Salamanca

**Keywords:** *Brucella* spp, brucellosis, epidemiology, fever of intermediate duration, malt fever, Mediterranean fever, Spain

## Abstract

Brucellosis remains one of the main zoonoses worldwide. Epidemiological data on human brucellosis in Spain are scarce. The objective of this study was to assess the epidemiological characteristics of inpatient brucellosis in Spain between 1997 and 2015. A retrospective longitudinal descriptive study was performed. Data were requested from the Health Information Institute of the Ministry of Health and Equality, which provided us with the Minimum Basic Data Set of patients admitted to the National Health System. We also obtained data published in the System of Obligatory Notifiable Diseases. A total of 5598 cases were registered. The period incidence rate was 0.67 (95% CI 0.65–0.68) cases per 100 000 person-years. We observed a progressive decrease in the number of cases and annual incidence rates. A total of 3187 cases (56.9%) came from urban areas. The group most at risk comprised men around the fifth decade of life. The average (±s.d.) hospital stay was 12.6 days (±13.1). The overall lethality rate of the cohort was 1.5%. The number of inpatients diagnosed with brucellosis decreased exponentially. The group of patients with the highest risk of brucellosis in our study was males under 45 years of age and of urban origin. The lethality rate has reduced to minimum values. It is probable that hospital discharge records could be a good database for the epidemiological analysis of the hospital management of brucellosis and offer a better information collection system than the notifiable diseases system (EDO in Spanish).

## Introduction

Brucellosis is an infectious disease caused by several species of facultative intracellular and slow-growing Gram-negative coccobacilli of the genus *Brucella* [1–3]. It is the main bacterial zoonosis in the world [1], and the main species responsible for human disease are *B. melitensis*, *B. abortus*, *B. suis* and *B. canis* [3]. *B. melitensis* is the most virulent species and the main causal agent of human brucellosis [3]. Three main biotypes of *B. melitensis* with different geographic distributions have been described [2]. The main forms of transmission of brucellosis to humans are the consumption of unpasteurised milk or derivatives (i.e. raw milk, soft cheese, butter and ice cream) from infected animals and contact with mucous membranes or inhalation of aerosols derived from infected animals [2]. Other less frequent forms of infection are those acquired in clinical laboratories as well as vertical and horizontal human-to-human transmission [3].

Human brucellosis is usually an acute systemic disease with an incubation period of 2–24 weeks [3]. Some patients may present relapses even after receiving treatment or develop chronic osteoarticular (peripheral arthritis, sacroiliitis and spondylitis), genitourinary (epididymo-orchitis), neurological (meningoencephalitis, meningovascular disease, brain abscesses and demyelinating syndromes), or endocarditis presentations [1–5]. A delay in diagnosis of more than 14 days significantly increases the rate of complications, estimated between 4% and 25% [1, 6]. As the clinical manifestations of brucellosis are not pathognomonic, the diagnosis is based on the use of direct microbiological studies (cultures or nucleic acid amplification tests) or indirect tests (serology) [7]. The treatment of brucellosis is based on the use of antimicrobials, usually in combination and prolonged [8, 9]. Mortality in adequately treated patients is minimal, although in complicated forms (i.e. endocarditis or meningoencephalitis), it can reach from 2% to 5%.

The actual incidence of human brucellosis is unknown, averaging 500 000 cases worldwide each year, although this figure is probably underestimated [6]. The incidence of human brucellosis is highly variable (0.02–268.81 per 100 000 person-years) depending on the country, being higher in the Middle East, central Asia and African Mediterranean rim and lower in central and southern Latin America, Western Europe (Greece, Italy and Spain) and North America [10–13]. Furthermore, the data are not uniform within each country and vary depending on the time period [11–13]. Epidemiological data on human brucellosis in Spain are scarce [14], so the objective of this study was to assess the epidemiological characteristics of inpatient brucellosis in Spain between 1997 and 2015.

## Patients and methods

This is a retrospective longitudinal descriptive study of hospitalised patients diagnosed with brucellosis in Spanish public hospitals between 1 January 1997 and 31 December 2015. This study analyses the data provided by hospital discharge records (HDRs). HDRs include all hospital discharges produced in the network of general hospitals in the National Health System (NHS). The data contained in this record are those established in the Hospitalisation Minimum Data Set (CMBD in Spanish). The CMBD is the main clinical-administrative database for knowledge of morbidity and the care process of patients treated in all public and private hospitals in Spain. It provides usual demographic data (age, gender, and place of residence), identifies the care provider (centre, unit), the patient (medical record number, health card number), clinical variables (diagnoses and procedures) and variables related to the episode of hospitalisation, as a circumstance of admission (urgent or scheduled), patient discharge (discharge to your address, transfer to another hospital or death), and average stay. Diagnoses and procedures collected are coded using the International Classification of Diseases, Clinical Modification (ICD-9-CM). *Principal diagnosis* was defined as the condition after study, which occasioned admission to the hospital, according to the ICD-9-CM Official Guidelines for Coding and Reporting. *Secondary diagnoses* (up to 13) are *‘other diagnoses’* or conditions that coexist at the time of admission or develop subsequently and that affect patient care during the current episode.

Our data were obtained from the Minimum Basic Data Set (CMBD in Spanish) of patients admitted to the NHS with ICD-9-CM diagnosis code 023-Brucellosis, provided by the Health Information Institute of the Ministry of Health and Equality. Patients with missing data were excluded from the study.

We also obtained data published in the System of Obligatory Notifiable Diseases (in Spanish, EDO, www.mscbs.gob.es), one of the information systems that integrates the National Network of Epidemiological Surveillance of Spain (in Spanish, RENAVE), which establishes the list of obligatory notifiable diseases, their notification modalities and the periodic diffusion of information in the Weekly Epidemiological Bulletin.

### Statistical analysis

Data analysis was performed using Statistical Package for the Social Sciences 26 (SPSS 26). Descriptive statistics were used to analyse the data initially. Categorical variables were summarised as frequencies (n) and percentages (%) and continuous variables as the mean, standard deviation (s.d.), median, interquartile range (IQR) (*Q*_3_–*Q*_1_), and range (minimum value, maximum value). For categorical variables, the odds ratio (OR) was used as a measure of association, and the 95% confidence interval (CI) of the OR was utilised to assess the precision of this estimate. Chi-square (*χ*^2^) test was used to assess the difference in proportions amongst subgroups and Student's *t*-test, the Mann−Whitney test and ANOVA test were applied to obtain the level of significance in continuous variables. Logistic regression model was applied to get the predicted category that had the maximum estimated probability and constructed cross tables to evaluate the accuracy of prediction classification (*B* coefficient is an odds ratio (OR = Exp(*B*)) and Wald *χ*^2^ test). The level of significance was expressed as *P*-values. A *P*-value <0.05 was considered statistically significant.

Incidence rates were computed by autonomous community and year to assess temporal and geographical patterns. The results in terms of mean rates by autonomous community were plotted on maps for the whole study period. The *incidence rate* was calculated by dividing the number of new cases of brucellosis (numerator) per year/period by the population at risk (denominator) in a period of time (person-years) multiplied by 100 000 and expressed as ‘cases per 100 000 person-years’. As it is not possible to accurately measure disease-free periods, the total person-time at risk can be estimated approximately and satisfactorily when the size of the population is stable, multiplying the average population size studied by the duration of the observation period. Thus, the population at risk was obtained from annual data published by the National Institute of Statistics (INE, http://www.ine.es/). The 95% confidence interval (95% CI) for the incidence rate was calculated for a better clinical application of the results. The *lethality rate* was calculated by dividing the number of deaths caused by a disease in a period and area (numerator) by the number of cases diagnosed for the same disease in the same period and area (denominator) (×100). It is the proportion of cases in a designated population of a particular disease, which die in a specified period of time. It is also known as *Case fatality rate*. Lethality is a better measure of clinical significance of the disease than mortality.

## Results

### Temporal and geographical distribution

**Fig. 1.**
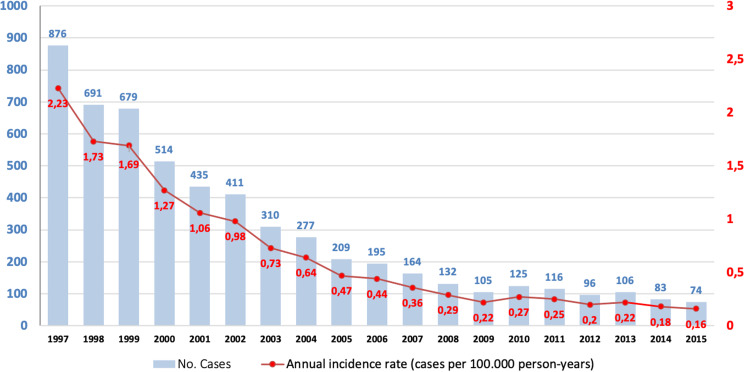
Temporal distribution of human brucellosis in Spain, 1997–2015: cases and annual incidence rate (cases per 100 000 person-years).

A total of 5598 cases with ICD-9-CM Diagnosis code 023 were registered in Spain during the 19-year study period, 1997–2015. The period incidence rate was 0.67 (95% CI 0.65–0.68) cases per 100 000 person-years. We observed a progressive decrease in the number of cases and annual incidence rates (Fig. 1), with the highest in 1997, 2.23 (95% CI 2.37–2.08) cases per 100 000 person-years (876 cases), and the lowest in 2015, 0.16 (95% CI 0.12–0.19) cases per 100 000 person-years (74 cases). When we compared the data recorded in the CMBD with the data reported in the EDO system, there were significant differences between the two health information systems (Table 1). At the beginning of the study period, more cases were recorded in the EDO system (2140 *vs.* 876), with a higher rate (5.45 *vs.* 2.23 cases per 100 000), while at the end of the study period, more cases were recorded in the CMBD (74 *vs.* 50), with a higher rate (0.16 *vs.* 0.11 cases per 100 000).

**Table 1.** Human brucellosis in Spain, 1997–2015: minimum basic data set (CMBD in Spanish) *vs.* system of obligatory notifiable diseases (EDO in Spanish)

Year	CMBD			EDO	
	No. of cases	Cases per million	Cases per 100 000	No. of cases	Cases per 100 000
1997	876	22.28	2.23	2140	5.45
1998	691	17.34	1.73	ND	ND
1999	679	16.89	1.69	ND	ND
2000	514	12.69	1.27	ND	ND
2001	435	10.58	1.06	ND	ND
2002	411	9.82	0.98	ND	ND
2003	310	7.26	0.73	ND	ND
2004	277	6.41	0.64	ND	ND
2005	209	4.74	0.47	ND	ND
2006	195	4.36	0.44	ND	ND
2007	164	3.63	0.36	ND	ND
2008	132	2.86	0.29	ND	ND
2009	105	2.25	0.22	ND	ND
2010	125	2.66	0.27	ND	ND
2011	116	2.46	0.25	104	0.22
2012	96	2.03	0.20	88	0.19
2013	106	2.25	0.22	104	0.22
2014	83	1.77	0.18	83	0.18
2015	74	1.59	0.16	50	0.11
TOTAL	5598	6.68	0.67	**–**	**–**

ND, No data.

**Fig. 2.**
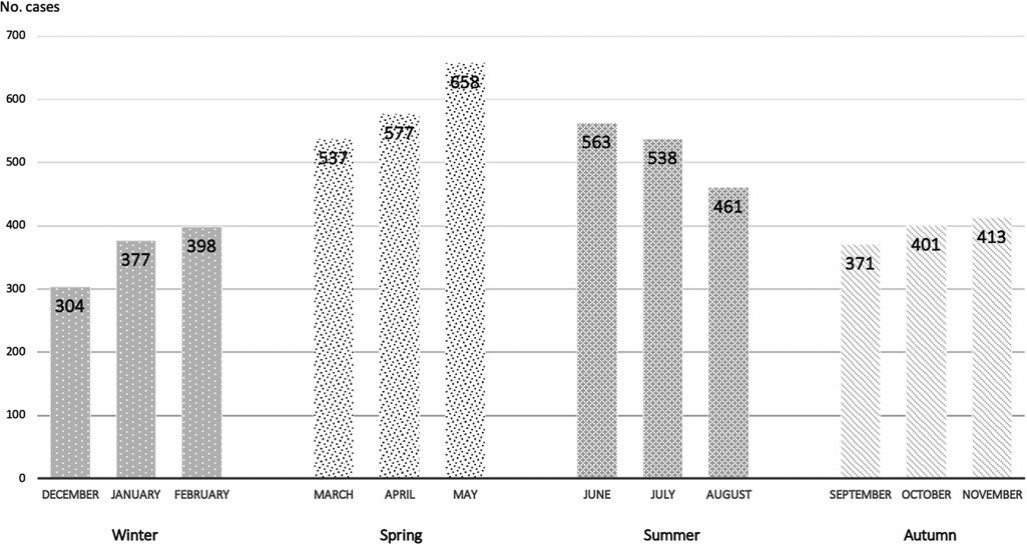
Distribution of brucellosis cases in Spain in the month of the year, 1997–2015.

The disease has a seasonal component, with a higher number of cases in the spring and summer months (from March to August), although there are cases throughout the year. The distribution of brucellosis cases in Spain during the months of the year is shown in Figure 2.

**Fig. 3.**
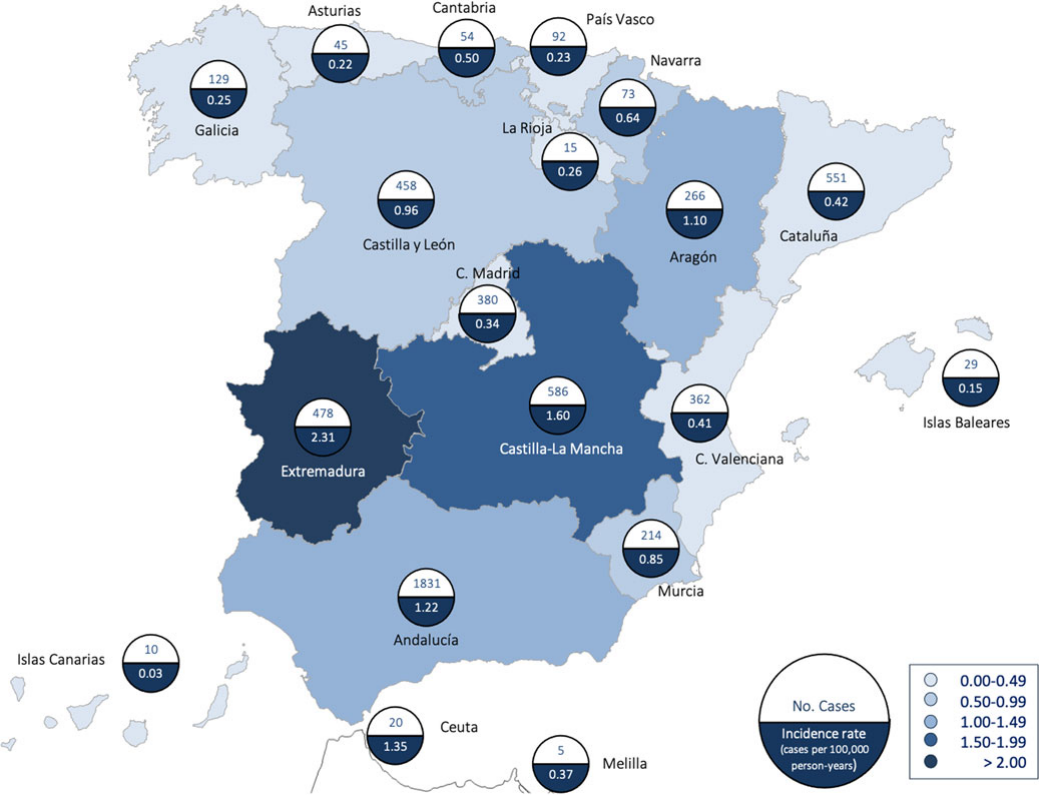
Number of cases and incidence rates (cases per 100 000 person-years) by regions, Spain, 1997–2015.

The geographical distribution of cases is shown in Figure 3. The highest incidence rates correspond to the central regions of the Iberian Peninsula: Extremadura, 2.31 (95% CI 2.10–2.52) cases per 100 000 person-years, and Castilla-La Mancha, 1.60 (95% CI 1.47–1.73) cases per 100 000 person-years. In contrast, Islas Canarias and Baleares, Cantabria and the Mediterranean coastal areas had the lowest incidence rates.

More than half of cases (56.9%; 3187) came from urban areas (population with more than 5000 inhabitants), compared to 29.2% (1637) from rural areas (population with less than 5000 inhabitants) (see Table 2). In some regions of Spain, cases of rural origin were more frequent than those of urban origin (*P* < 0.001): Cantabria (63.8% *vs.* 36.2%), Castilla y León (63% *vs.* 37%), Navarra (61.2% *vs.* 38.8%), and Extremadura (60.2% *vs.* 39.8%). On the other hand, other autonomous communities had a higher number of patients of urban origin: Andalucía (72.2% *vs.* 27.8%), Galicia (79.5% *vs.* 20.5%), Cataluña (83.7% *vs.* 16.3%), C. Valenciana (87.3% *vs.* 16.3%), País Vasco (96.6% *vs.* 3.4%), Murcia (97.4% *vs.* 2.6%), and Madrid (98% *vs.* 2%). No significant differences were observed associating rural/urban origin with seasonality (*P* = 0.884).

**Table 2.** Main demographic and clinical data of Brucellosis inpatients in Spain from 1997 to 2015

Variables	*N* = 5598 cases (100%) *n* (%)
Age (years)	
Mean ± s.d.	45.8 ± 21.1
Range (minimum value, maximum value)	(0, 101)
Age 0–14 years	476 (8.5)
Age 15–44 years	2183 (39.0)
Age 45–64 years	1657 (29.6)
Age ≥65 years	1282 (22.9)
Gender	
Male	4131 (73.8)
Female	1465 (26.2)
Undetermined	2 (0.0)
Rural *vs.* urban environment	
Rural (population with more than 5000 inhabitants)	1637 (29.2)
Urban (population with less than 5000 inhabitants)	3187 (56.9)
Foreigners	17 (0.3)
Unknown	757 (13.5)
ICD-9-CM: 023 code	5598 (100.0)
*Brucella melitensis* (023.0)	365 (6.5)
*Brucella abortus* (023.1)	24 (0.4)
*Brucella suis* (023.2)	5 (0.1)
*Brucella canis* (023.3)	2 (0.0)
Other (023.8)	211 (3.8)
Unspecifed (023.9)	4991 (89.2)
Diagnosis causing the hospitalisation	
Principal diagnosis	3767 (67.3)
Secondary diagnosis	1831 (32.7)
Type of hospital admission	
Urgent	4624 (82.6)
Programmed	965 (17.2)
Others/unknown	9 (0.2)
Type of discharge	
Home	5337 (95.3)
Transfer to another hospital	118 (2.1)
Transfer to social-health center	4 (0.1)
Voluntary discharge	25 (0.4)
Others/unknown	30 (0.5)
Overall lethality	84/5598 (1.50)
Brucellosis principal diagnosis lethality	12/3755 (0.32)
Hospital stay (days)	
Mean ± s.d.	12.6 ± 13.1
Range (minimum value, maximum value)	(0, 194)

**Fig. 4.**
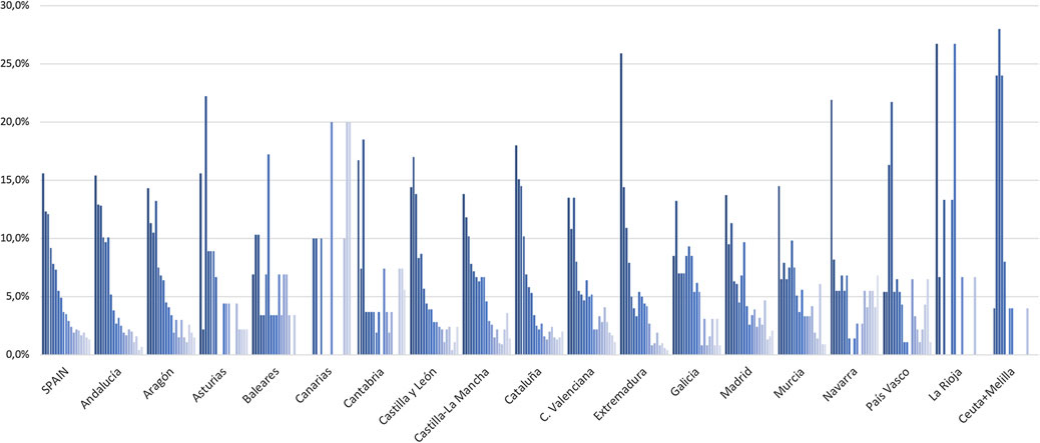
Annual percentage distribution of brucellosis cases by autonomous communities of Spain in the study period, 1997–2015.

Annual evolution of brucellosis cases in each region (autonomous community) of Spain is analysed in Figure 4. There are significant differences (*P* < 0.001) in the percentage distribution of the cases annually. The global profile of Spain varied throughout the entire period, being most striking in the Islas Canarias, Cantabria, La Rioja and Melilla.

### Distribution by gender and age

The number of cases in men (73.8%) was three times higher than that in women (26.2%), with a male/female ratio of 3:1 (4131/1465): incidence rate in men, 1.00 (95% CI 1.03–0.97) cases per 100 000 person-years *vs.* In women, 0.34 (95% CI 0.32–0.36) cases per 100 000 person-years. The mean (±s.d.) age was 45.8 years (± 21.1) (median (IQR), 46.4 (63–15)), range (0, 101). A total of 8.5% (476 cases) of the sample corresponded to the paediatric population (0–14 years), with 338 (43.3%) adults (15–64 years) and 110 (14.1%) elderly patients (Table 2).

There were statistically significant differences between men and women in the percentage distribution of cases by decades of age, as shown in Table 3 (*P* < 0.001). Thus, the highest percentages in men were in those from 30 to 59 years old, while in women, they were in those 60−79 years old. In addition, the percentage of men of rural origin was slightly higher than that of urban origin, 78.9% *vs.* 71.3% (*P* = 0.015); the percentage of patients over 45 years of age of rural origin was slightly higher than that of urban origin, 55.7% *vs.* 52.8% (*P* = 0.058). There were no significant differences in seasonality among the age groups (*P* = 0.547).

**Table 3.** Patient cohort description according to age groups and gender

Age groups, years	Total, *N* = 5596 *n* (%)	Male, *N*_1_ = 4131 *n* (%)	Female, *N*_2_ = 1465 *n* (%)	*P*-value
0–9	243 (4.3)	146 (3.5)	97 (6.6)	*P* < 0.001
10–19	460 (8.2)	331 (8.0)	128 (8.7)	
20–29	715 (12.8)	540 (13.1)	174 (11.9)	
30–39	831 (14.8)	648 (15.7)	183 (12.5)	
40–49	808 (14.4)	656 (15.9)	152 (10.4)	
50–59	829 (14.8)	635 (15.4)	194 (13.2)	
60–69	863 (15.4)	626 (15.2)	237 (16.2)	
70–79	613 (11.0)	407 (9.9)	206 (14.1)	
80–89	212 (3.8)	128 (3.1)	84 (5.7)	
>90	24 (0.4)	14 (0.3)	10 (0.7)	

### Clinical data

Hospitalisations with ICD-9-CM Diagnosis code 023 as the principal diagnosis code represented 3767 (67.3%), with 1831 (32.7%) cases as secondary diagnosis code. Most cases (4991, 89.2%) were coded as *unspecified brucellosis:* ICD-9-CM Diagnosis code 023.9, as shown in Table 2.

Table 4 compares patients with principal diagnosis *vs.* secondary diagnosis. The mean age of patients with a principal diagnosis code was lower than that of those with a secondary diagnosis code (mean ± s.d., 41.5 ± 20.2 *vs.* 54.7 ± 20.1, *P* < 0.001). Additionally, average hospital stays increased by 3 days among patients with a secondary diagnosis code (mean ± s.d., 11.6 ± 10.5 *vs.* 14.8 ± 17.1, *P* < 0.001).

**Table 4.** Principal diagnoses code *vs.* secondary diagnoses code: (a) bivariate analysis, (b) multivariate analysis

Variables	Principal diagnosis *N*_1_ = 3767 *n* (%)	Secondary diagnosis *N*_2_ = 1831 *n* (%)	*P*-value	OR (95% CI for OR)
(a) Bivariate analysis				
Age (years), mean ± s.d.	41.5 ± 20.2	54.7 ± 20.1	<0.001	
Age 0–14 years	420 (11.1)	56 (3.1)	<0.001	
Age 15–44 years	1682 (44.7)	501 (27.4)		
Age 45–64 years	1064 (28.2)	593 (32.4)		
Age ≥65 years	601 (16.0)	681 (37.2)		
<45 years	2102 (55.8)	557 (30.4)	<0.001	2.9 (2.5–3.2)
≥45 years	1665 (44.5)	1274 (69.6)		
Gender				
Male	2831 (75.2)	1300 (71.0)	0.001	1.2 (1.1–1.4)
Female	934 (24.8)	531 (29.0)		
Origin of cases				
Rural	1057 (33.4)	580 (35.0)	0.239	0.9 (0.8–1.1)
Urban	2112 (66.6)	1075 (65.0)		
Type of hospital admission				
Urgent	3264 (86.6)	1360 (74.3)	<0.001	2.2 (1.9–2.5)
Programmed	498 (13.2)	467 (25.5)		
Type of discharge				
Home	3668 (97.4)	1669 (91.2)	<0.001	3.6 (2.8–4.6)
Others	99 (2.6)	162 (8.8)		
Exitus letalis	12 (0.3)	72 (3.9)	<0.001	0.08 (0.04–0.14)
Hospital stay (days), mean ± s.d.	11.6 ± 10.5	14.8 ± 17.1	<0.001	

(b) Multivariate analysis: binary logistic regression model (dependent variable: principal diagnosis *vs.* secondary diagnosis)								
Independent variables	*B*	Standard error	Wald	gl	Sig.	Exp(B)	95% CI for Exp(B)	
							Lower	Upper
Age	0.938	0.062	227.903	1	0.000	2.554	2.261	2.885
Gender	0.186	0.067	7.603	1	0.006	1.205	1.055	1.375
Type of hospital admission	0.690	0.075	84.009	1	0.000	1.994	1.720	2.311
Hospital readmission	0.872	0.105	69.057	1	0.000	2.392	1.948	2.939
Type of discharge	0.742	0.160	21.459	1	0.000	2.101	1.535	2.876
Exitus letalis	−1.510	0.355	18.075	1	0.000	0.221	0.110	0.443
Constant	−1.855	0.198	87.415	1	0.000	0.156		

Categorical variables were analysed using a logistic regression model. All independent variables included in the multivariate model were significantly associated (*P* < 0.05) with the dependent variable: principal diagnosis *vs.* secondary diagnoses, except for the rural or urban origin of the patients. The coefficients were positive (risk factor) and significant for the variables age, gender, type of hospital admission and type of discharge. Therefore, a logistic regression model allowed us to predict that a principal diagnosis is associated with a higher probability of a male patient, under 45 years of age, with urgent admission and home discharge but a lower probability of death/failure. In relation to the type of hospital admission, 4624 (82.6%) cases were urgent. Most cases (5337, 95.3%) were sent home after hospital discharge. The average (±s.d.) hospital stay was 12.6 days (± 13.1) (median (IQR), 9 (15–5)) (see Table 2).

We do not know the service responsible for the patient's discharge in three quarters of the sample. Of the remaining 25% in which the responsible service was known, half of them (718, 12.8%) were treated in the Internal Medicine Service, followed by the Paediatric Service (62, 1.1%).

### Cohort lethality

The overall lethality rate of the cohort was 1.50 per 100 (84 deaths/5598 total). The principal diagnosis lethality rate for brucellosis was 0.32 per 100 (12 deaths/3755 total principal diagnoses). The highest annual brucellosis principal diagnosis lethality rate was 3.17% in 2008, decreasing to 0% since then. The lethality rate in males was 1.43 per 100 (59 deaths/4131 total males) and 1.71 per 100 females (25 deaths/1465 total females). Lethality rates varied according to age: 0–14 years (0 deaths/476); 15–44 years, 0.32 per 100 (7 deaths/2183 cases); 45–64 years, 1.15 per 100 (19 deaths/1657 cases); and >65 years, 4.52 per 100 (58 deaths/1282 cases). The lethality rate was 1.22 per 100 (20 deaths/1637 cases) in rural environments and was 1.88 per 100 (60 deaths/3187 cases) in urban patients.

## Discussion

During the study period, 5598 hospital admissions for brucellosis were registered, which represents an incidence rate of 0.67 cases per 100 000 inhabitants per year. Taking into account that the worldwide incidence ranges between 0.02 and 268.81 per 100 000 person-years in endemic areas [11–13], Spain is at the lower limit. However, Spain has a clearly higher rate than other endemic areas, such as Australia and China [6, 15], but a lower rate than other European countries, such as Greece and Italy [11]. However, the data obtained in hospitalised patients do not include asymptomatic infections [16], so in some countries, the incidence of brucellosis may be underestimated by 12–18 times [17].

The evolution of human brucellosis incidence rates in Spain from 1997 until 2015 has decreased progressively, and there are indirect data on the progressive decline since then. There are several non-exclusive explanations for these data: (i) the source of information (CMBD *vs.* EDO). This decrease is more significant in the EDO notification system (5.45 cases to 0.11 cases per 100 000). These data are justified by the methodological differences in data collection; thus, while the CMBD is a mandatory record for hospitals in our NHS, the EDO system is a reporting system based on the ethical responsibility of health professionals. For this reason, it is likely that the CMBD is a more reliable information collection system than the EDO system for inpatients. (ii) The establishment of control programmes [18]. (iii) The better knowledge of the disease that implies an earlier diagnosis and a more effective treatment. The results between the different registry systems suggest the need for unique quality registries.

The incidence of brucellosis varies widely not only among countries but also among different regions of the same country. In Spain, the highest incidence is observed in interior regions (Extremadura and Castilla la Mancha), similarly to the situation in other countries (i.e. China) [15] but different from that in others (i.e. Australia) [6]. However, it is extremely low in some regions (i.e. Canary Islands), which suggests an imported origin of the infection in these areas [19]. These differences suggest that demographic, occupational, and socioeconomic factors may play a role. In Spain, slightly more than half of the cases come from urban areas, although the limit used in the definition of rural or urban areas is somewhat arbitrary, which may explain the similarity with some series [20] and the differences with others [6]. In general, the most affected areas are those least economically developed and/or with the highest livestock density (sheep and goats) [10].

In this study, the number of cases in men was three times higher than that in women. This finding is similar to the results obtained in agricultural areas and different from those described in livestock areas [20]. The average age was 45.8 years, similar to that reported in other studies [20]. When the incidence relationship with both magnitudes was evaluated, it was observed that it was higher in younger men (30–59 years) and older women (60–79 years), an aspect not described, to our knowledge, in other series. In our study, the annual period with the highest number of cases covers from March to July, which is similar to other series [20].

The fact that brucellosis has been identified as the primary diagnosis in more than 67% of cases indicates a much higher diagnostic suspicion than in other infections that cause fever of intermediate duration [21, 22]. The mean stay of individuals with a secondary diagnosis is significantly longer than that in the primary forms. This result seems quite logical, since there is a longer delay in time to diagnosis and, predictably, a greater number of complications that prolong the stay; predictably, a greater number of complications prolong the stay.

One of the main limitations of this study is that we do not know the diagnostic method applied individually. Thus, secondary diagnoses could be due to a positive serological test, and be both an acute infection and a ‘serological sequel’ of an old infection.

In this series, the mortality of brucellosis cases is clearly lower than that in the literature, being zero since 2008, although it increases with age (*P* < 0.001). This could be due to being an endemic area where the diagnostic suspicion is greater than that in other areas. In our series, we found a higher lethality rate in women than in men, and these data are probably biased because the cohort of women is older than the cohort of men. We also detected that in urban cases, the lethality rate is higher than in rural cases.

The CMBD is a standardised registry of patients that is carried out in most of the hospitals in our country and is therefore not exposed to the biases that limit other types of registries that involve voluntary declaration. This aspect gives it two fundamental advantages: the first is that the number of individuals who make up the sample is very large, and the second is that such a large sample n makes it very representative of the population. However, the design of the CMBD has limitations, such as the absence in the registry of patient comorbidities, clinical manifestations, the results of complementary tests and the therapeutic measures used.

In summary, inpatient diagnosis for brucellosis decreased exponentially in the study period in Spain, probably due to success of veterinary control programmes and/or an earlier diagnosis and treatment. The highest incidence rates corresponded to the central and interior regions of Spain, and the group of patients with the highest risk of suffering from brucellosis in our study by logistic regression was males under 45 years of age and of urban origin. The lethality rate has also been reduced to minimum values. It is probable that HDRs could be a good database for the epidemiological analysis of the hospital management of brucellosis and offer a better information collection system than the EDO system.

### Ethical approval

This study is based on medical data of patients collected in the CMBD. These data are the responsibility of the Ministry of Social Services of Health and Equality (*Ministerio de Servicios Sociales, Sanidad e Igualdad*, MSSSI) that collects and organises them. All patient data provided by the CMBD are anonymised and deidentified by the MSSSI before they are provided to applicants. According to this confidentiality commitment signed with the MSSSI, researchers cannot provide the data to other researchers, so other researchers must request the data directly from the MSSSI. The protocol and ethics statement of this study were approved by the Clinical Research Ethics Committee of the Complejo Asistencial Universitario de Salamanca (CAUSA-Salamanca, Spain CEIMc) with the assigned Code PI 2021 03 708. Because the data were obtained from an epidemiological database, written consent was not obtained. All data analysed were anonymised.

### Consent to participate

Because the data were obtained from an epidemiological database, written consent was not obtained. All data analysed were anonymised.

### Consent to publish

All authors consent to publish.

## Availability of data and materials

Available in public deposits in www.mscbs.gob.es.
